# Effect of Myricetin on the Prevention of Noise-Induced Hearing Loss-An Animal Model

**Published:** 2019-09

**Authors:** Maryam Bahaloo, Mohammad Ebrahim Rezvani, Ehsan Farashahi Yazd, Mohammad Hossein Davari, Amir Houshang Mehrparvar

**Affiliations:** 1 *Industrial Diseases Research Center, Shahid Sadoughi University of Medical Sciences, Yazd, Iran. *; 2 *Department of Physiology, Shahid Sadoughi University of Medical Sciences, Yazd, Iran.*; 3 *Stem Cell Biology Research Center, Yazd Reproductive Sciences Institute, Shahid Sadoughi University of Medical Sciences, Yazd, Iran.*; 4 *Department of Occupational Medicine, Shahid Sadoughi University of Medical Sciences, Yazd, Iran. *

**Keywords:** Antioxidant, Myricetin, Noise-Induced hearing loss, Prevention

## Abstract

**Introduction:**

Exposure to hazardous noise induces one of the forms of acquired and preventable hearing loss that is noise-induced hearing loss (NIHL). Considering oxidative stress as the main mechanism of NIHL, it is possible that myricetin can protect NIHL by its antioxidant effect. Therefore, the present study aimed to investigate the preventive effect of myricetin on NIHL.

**Materials and Methods:**

A total of 21 Wistar rats were randomly divided into five groups, namely (1) noise exposure only as control group, (2) noise exposure with the vehicle of myricetin as solvent group, (3) noise exposure with myricetin 5 mg/kg as myricetin 5 mg group, (4) noise exposure with myricetin 10 mg/kg as myricetin 10 mg group, (5) and non-exposed as sham group. The hearing status of each animal was assessed by Distortion Product Otoacoustic Emissions.

**Results:**

The levels of response amplitude decreased after the exposure to noise in all groups and returned to a higher level after 14 days of noise abstinence at most frequencies; however, the difference was not significant in the myricetin-receiving or control groups.

**Conclusion:**

The results of this study showed that two doses of myricetin (5 and 10 mg/kg) administered intraperitoneally could not significantly decrease transient or permanent threshold shifts in rats exposed to loud noise.

## Introduction

Noise is one of the most common hazards in the occupational environment (1). Exposure to hazardous noise induces one of the forms of acquired and preventable hearing loss that is noise-induced hearing loss (NIHL) (2,3). It is estimated that 12% of the world population is at risk of NIHL (4). According to the World Health Organization reports, NIHL is the cause of one-third of hearing loss worldwide, and the incidence of this disorder is increasing (5,6). This type of hearing loss is usually bilateral and symmetric (7).Noise exposure can damage the inner ear by two mechanisms, namely direct mechanical trauma and metabolic damage to the organ of Corti in which the formation of reactive oxygen species (ROS) is a part of this mechanism (8). Acoustic overexposure increases ROS production in the inner ear, which eventually may damage hair cells. Other mechanisms, such as a decrease in glutathione peroxidase activity in the inner ear, may have a role as well (9). 

Noise damage depends on the noise intensity, frequency, and duration of exposure and may lead to temporary threshold shift (TTS) or permanent threshold shift (PTS) (10,11). Today, the treatment of hair cell damage is limited and includes hearing aid, as well as cochlear implantation, which is expensive and not accessible in many regions (12,13). Therefore, investigations continue to find ways for the prevention of NIHL. The primary method of prevention is to seek early findings of NIHL before clinical disease, using screening methods, such as pure-tone audiometry or otoacoustic emissions (OAEs). Recently, using pharmacological agents is also proposed for the prevention of NIHL (3).

In recent studies, there is a focus on the essential role of ROS and using antioxidants for the prevention of NIHL (13-15). 

In animal and human studies, the antioxidants, such as vitamins C and E, Q10, resveratrol, ferulic acid, N-acetylcysteine, and glutamate receptor (N-methyl-D-aspartate) antagonists have been observed to have protective effects against noise trauma (16-19). Recently, clinical trials have been performed using different substances, such as Ebselen, for the prevention and treatment of NIHL (20).

Myricetin (3,5,7,3',4',5'-hexahydroxyflavone) is a natural flavonoid derivated from fruit vegetables and berries (21,22). It has been shown that this substance has anticarcinogenic, antimicrobial, antithrombotic, antidiabetic (hypoglycemic component), as well as antioxidant and cytoprotective effects (21-24). Myricetin can scavenge ROS strongly and inhibit DNA damage (22,24,25). The antioxidant effect of myricetin and ROS scavenging properties due to its active hydroxyl groups can probably inhibit NIHL because one of the main mechanisms of inducing NIHL is oxidant stress (8). Myricetin has been used with different doses and routes of administrations in order to assess its antioxidant effect. Ramezani et al. demonstrated beneficial effects of myricetin intraperitoneally administered in 5mg/kg and 10 mg/kg doses in rats for the prevention of oxidative stress in neuronal pathways (26). Moreover, Sun et al. showed the beneficial anti-ischemic effect of 5mg/Kg myricetin administered intraperitoneally to rats (27). Considering oxidative stress as the main mechanism of NIHL, it is possible that myricetin can protect NIHL by its antioxidant effect; Therefore, the present study aimed to investigate the preventive effect of myricetin on NIHL. 

## Materials and Methods

The present laboratory experimental study was performed on Wistar rats in Shahid Sadoughi University of Medical Sciences, Yazd, Iran, to detect whether myricetin can prevent NIHL. This is the report of the first part of the study (i.e., electrophysiological assessment of NIHL). 

**Animals**

Male adult Wistar rats (average weight: 250-275 g, 2 months old) were obtained from the Animal Center of Shahid Sadoughi University of Medical Sciences. The animals were kept in a room with a temperature of 22-23°C, under a 12 h light/dark cycle with free access to food and water. 

All procedures were performed in the Central Laboratory of Medical School of Shahid Sadoughi University of Medical Sciences in 2017. The protocol of the study was approved by the University Ethics Committee. All efforts were made to reduce the number of animals used and minimize their suffering according to the national codes of research on animals.

**Experimental groups**

A total of 21 Wistar rats were randomly divided into five groups (using random number table), including 1) noise exposure only as control group (n=6; 12 ears), 2) noise exposure with the vehicle of myricetin (ethylic alcohol 5%) as solvent group (n=4; 8 ears), 3) noise exposure with myricetin 5 mg/kg as myricetin 5 mg group (n=4; 8 ears), 4) noise exposure with myricetin 10 mg/kg as myricetin 10 mg group (n=4; 8 ears), and 5) non-exposed as sham group (n=3; 6 ears). The last three groups of animals started receiving the drug or vehicle for 3 days before noise exposure and then 1 h before noise exposure for 10 days once a day. The drug and vehicle were delivered by intraperitoneal injection (26,27). Myricetin was purchased from Sigma-Aldrich (70050-100 MG, Sigma-Aldrich Chemie GmbH, Germany).

**Noise exposure**

All four groups were exposed to noise. The animals were exposed to a 10 kHz octave band noise of 100 dB sound pressure level for 1 h each day for 10 consecutive days in a glass cage. This exposure protocol was selected according to a study conducted by Fetoni et al. (15). The noise was generated by an audiometer (OB 929, Madsen, Denmark). Noise intensity was measured by a sound level meter (TES-1351 digital sound level meter, Taiwan).

*Hearing Assessment*

The hearing status of each animal was assessed by Distortion Product Otoacoustic Emissions (DP-OAEs) by Capella (Madsen, Denmark) under mild anesthesia (using ketamine 90 mg/kg and xylazine 10 mg/kg through intraperitoneal injection). Two probes were placed in the animal’s external canal of the ear. The DP-OAEs amplitudes were recorded at the frequencies of 2, 3, 4, 6, and 8 kHz in each ear. The DP-OAEs were performed for all animals on four occasions, including before treatment and noise exposure, 1 h after the first noise exposure, 1 h after the last noise exposure, and 14 days after the last noise exposure.

**Statistical Analysis**

All the data were analyzed by SPSS (version 20). P-value less than 0.05 was considered statistically significant. The results of the Kolmogorov-Smirnov test showed that the data distribution was not normal; therefore, nonparametric tests, including the Mann-Whitney U test and Kruskal-Wallis test were used for data analysis.

**Ethical considerations**

The present study was extracted from a PhD thesis in Occupational Health and approved by the Ethics Committee of Shahid Sadoughi University of Medical Sciences. All the ethical codes of the study on animals were considered in the present study.

## Results

Normal distribution was not observed in terms of the study variables; therefore, nonparametric tests were used to analyze the data. There was no significant difference in baseline hearing threshold among the five groups (P<0.001). The hearing thresholds increased significantly in all the groups at all frequencies after the exposure to noise, and there was a significant difference between baseline thresholds (i.e., the first measurement) and hearing thresholds 1 h after the first exposure (i.e., the second measurement) and 1 h after the last exposure (i.e., the third measurement) both showing TTS, and 14 days after the last exposure (i.e., the fourth measurement), which demonstrated PTS. 

**Fig 1:**
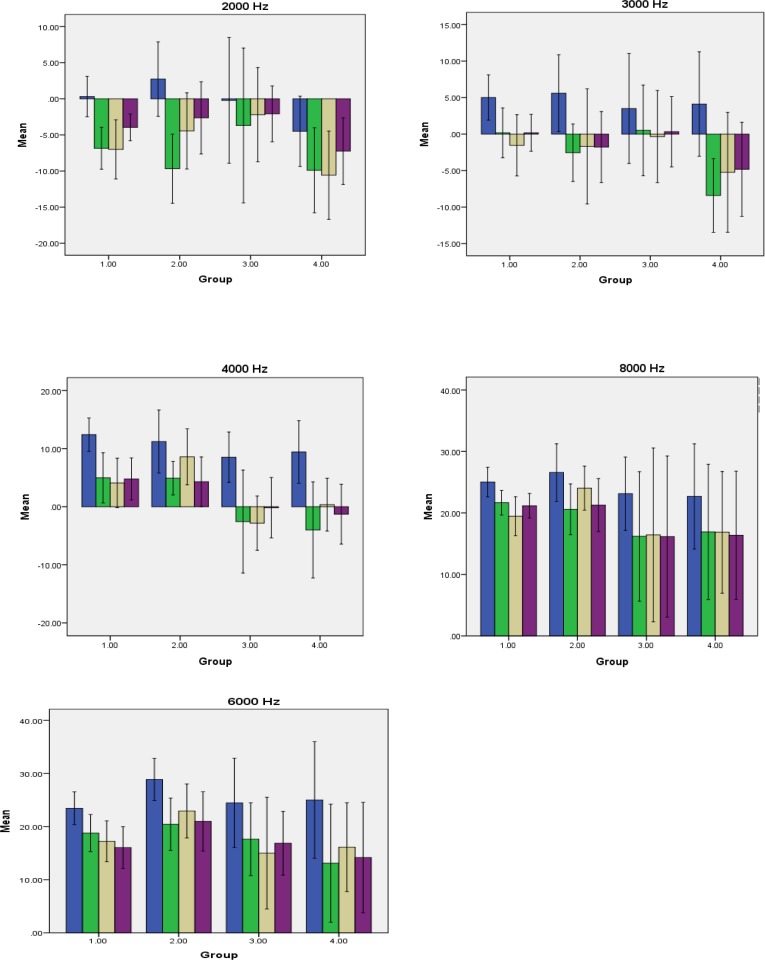
Means of response amplitude levels at different frequencies; columns demonstrating different measurement times (i.e., first: baseline; second: 1 h after first exposure; third: 1 h after last measurement; fourth: 14 days after last measurement); error bars: 95% confidence interval; group 1: control, group 2: solvent, group 3: 5 mg/kg myricetin, and group 4: 10 mg/Kg myricetin

Response amplitude levels decreased after the exposure to noise in all the groups. Figure 1 depicts comparing the means of response amplitudes among the groups at different frequencies and different times (i.e., before exposure, 1 h after the first exposure, 1 h after the last exposure, and 14 days after the last exposure). Response amplitude level returned to a higher level after 14 days of noise abstinence at most frequencies (group 1: 2, 3, 4, and 8 kHz, group 2: 2 and 3 kHz, group 3: 2, 4, and 8 kHz, and group 4: 2, 3, and 4 kHz); however, the difference was not significant in the myricetin-receiving groups and control groups.

Table 1 tabulates the comparison of mean differences of amplitude response levels after the exposure to noise between different groups at different occasions of the study. The difference in response amplitude level was significant only between groups 2 and 3, as well as 2 and 4, 1 h after the first exposure at 2 kHz, between groups 1 and 4, as well as 2 and 3, 1 h after the first exposure at 3 KHz, and between groups 2 and 3, as well as 2 and 4, 1 h after the last exposure at 4 KHz.

**Table 1:** P-value of one by one comparison of mean difference of response amplitude levels after exposure to noise at different measurement times between groups (italic fonts suggestive of significant difference)

**Hearing frequencies (Hz)**	Time of measurement*	Comparisons between groups**			
		**1 vs. 3**	**1 vs. 4**	**2 vs. 3**	**2 vs. 4**
2000	Temporary threshold shift 1	0.07	0.62	*0.01*	*0.04*
	Temporary threshold shift 2	0.11	0.73	0.16	0.79
	Permanent threshold shift	0.35	0.42	0.19	0.16
3000	Temporary threshold shift 1	0.19	*0.01*	*0.04*	0.28
	Temporary threshold shift 2	0.12	0.57	0.44	0.51
	Permanent threshold shift	0.39	0.31	0.23	0.72
4000	Temporary threshold shift 1	0.73	0.34	0.64	0.19
	Temporary threshold shift 2	0.24	0.62	*0.01*	*0.01*
	Permanent threshold shift	0.68	0.27	0.88	0.19
6000	Temporary threshold shift 1	0.18	0.06	0.72	0.40
	Temporary threshold shift 2	0.38	0.73	0.28	0.46
	Permanent threshold shift	1.00	0.18	0.96	0.40
8000	Temporary threshold shift 1	0.24	0.52	0.72	0.83
	Temporary threshold shift 2	1.00	0.52	0.13	0.29
	Permanent threshold shift	0.79	0.62	0.72	0.71

* Temporary threshold shift (TTS) 1: mean difference of response amplitude level between baseline and 1 h after first exposure; TTS 2: mean difference of response amplitude level between baseline and 1 h after last exposure; permanent threshold shift: mean difference of response amplitude level between baseline and 14 days after last exposure

** Study groups: 1 (control); 2 (solvent); 3 (myricetin 5 mg); 4 (myricetin 10 mg)

## Discussion

Noise is one of the stressors in most workplaces. Long time exposure to loud noise causes NIHL (28). The NIHL is one of the most common forms of hearing loss, which is caused due to the injury in cochlea and structures of the inner ear and leads to a form of sensorineural hearing loss (29). One of the most important mechanisms causing NIHL is the production of ROS after the exposure to noise, which may lead to cell death. Therefore, new interventions try to scavenge ROS using various antioxidants (30).

Different antioxidants have been assessed for their protective effects on NIHL with different results. 

In this study, a different antioxidant that is myricetin was used to assess its protective effects on NIHL in rats. Myricetin is a natural flavonoid present in tea and different kinds of mulberries and can decrease oxidative stress due to aging (23,31). The results of this study showed that two doses of myricetin (5 and 10 mg/kg) administered intraperitoneally could not significantly decrease TTS or PTS in rats exposed to loud noise. 

Vitamin C as a potent antioxidant could lower the incidence of TTS exposed to noise (32). Oral administration of ACUVAL 400®, a food supplement multivitamin containing various vitamins (e.g., A, E, B1, B2, B6, and B12), L-Arginine, Ginkgo Biloba, and minerals, such as magnesium, selenium, and zinc, as well as small amounts of Q10, could improve hearing threshold in rats exposed to loud noise (33). Other substances, such as silymarin, N-acetyl cysteine, glutamate receptor antagonists, Ebselen, and statins, were also effective in the prevention of NIHL (9,19,34-37). In addition, N-acetyl cysteine with salicylate and D-methionine were effective in the prevention of hair cell loss after the exposure to noise (38,39), although Davis et al. did not observe a protective effect for N-acetyl cysteine in PTS (40). Moreover, Pourbakht et al. could not identify a protective effect for celecoxib in guinea pigs (41). 

 No studies were detected regarding assessing the preventive effect of myricetin, especially on NIHL, although positive anticytotoxic and antioxidant effects of myricetin have been shown in previous studies on the prevention of injuries in disorders other than NIHL (21,26,27). The present study failed to show a significant effect for myricetin when administered intraperitoneally on the prevention of NIHL in rats. Choi et al. compared the effect of oral and intraperitoneal administration of N-acetyl cysteine and 4-hydroxy alpha-phenyl-tert-butyl nitrone and find that oral administration had significantly higher preventive effects on NIHL in chinchillas (42). Therefore, performing further investigations using the oral route to administer myricetin may be of importance in assessing the preventive effect of this substance on NIHL. 

 In the present study, there were some limitations. Acoustic brainstem responses could not be used to assess the hearing status of the rats due to financial constraints; therefore, OAEs were used that is not considered a gold standard test. In addition, only two doses of myricetin (5 and 10 mg/kg) were used via intraperitoneal administration. Further studies with different (i.e., lower or higher) doses and other routes of administration (i.e., oral or intravenous) could be performed in the future leading to different results. Another limitation of this study was the lack of statistical power. Studies with larger sample size and higher power may probably show the preventive effect of this substance on NIHL. 

## Conclusion

The intraperitoneal administration of 5 and 10 mg/kg myricetin could not prevent NIHL in rats exposed to 100 dB noise for 10 days. 
